# Draft Genome Sequence of *Acholeplasma laidlawii*, a Common Contaminant of Cell Cultures

**DOI:** 10.1128/genomeA.01578-16

**Published:** 2017-02-02

**Authors:** Franciele Maboni Siqueira, Samuel Paulo Cibulski, Thais Fumaco Teixeira, Fabiana Quoos Mayer, Paulo Michel Roehe

**Affiliations:** a Departamento de Patologia Clínica Veterinária, Faculdade de Veterinária, Universidade Federal do Rio Grande do Sul, Porto Alegro, Rio Grande do Sul, Brazil; b Departamento de Microbiologia Imunologia e Parasitologia, Laboratório de Virologia, Universidade Federal do Rio Grande do Sul, Porto Alegro, Rio Grande do Sul, Brazil; c Laboratório de Biologia Molecular, Instituto de Pesquisas Veterinárias Desidério Finamor (IPVDF), Fundação Estadual de Pesquisa Agropecuária Fundação Estadual de Pesquisa Agropecuária, Porto Alegro, Rio Grande do Sul, Brazil

## Abstract

*Mollicutes* are important cell culture contaminants which may eventually affect the results of biological assays or affect their interpretation. *Acholeplasma laidlawii* is one of the most frequent contaminants of cell cultures. Here, we report the complete genome sequence of *A. laidlawii* strain MDBK/IPV, recovered from Madin-Darby bovine kidney (MDBK) cells.

## GENOME ANNOUNCEMENT

*Acholeplasma* species comprise bacteria from the *Mollicutes* class that are described as saprophyte and commensal bacteria. It has been suggested that acholeplasmas and phytoplasmas come from an *Acholeplasma*-like last-common ancestor (1). Unlike other mycoplasmas, members of the family *Acholeplasmataceae* do not require sterols for cultivation and are able to synthesize fatty acids from precursors (2). Some mycoplasmal species, among them *Acholeplasma laidlawii*, could persist as culture contamination on cell lineages and consequently can affect the results of biological assays or affect their interpretation. Moreover,* A. laidlawii* infects plants, with phytopathogenic effects analogous to *Phytoplasma* infection; besides, these bacteria were detected as commensals in insects (3). To our knowledge, only one complete genome sequence of *A. laidlawii* (strain PG-8A) has been published (4). The analysis of this *A. laidlawii* genome revealed a richly equipped repertoire for metabolism, SOS response, repair systems, and extensive transcriptional regulation machinery, including the two-component systems, riboswitches and T-boxes (4, 5). Here, we report the complete genome sequence of *A. laidlawii* strain MDBK/IPV, recovered from a Madin-Darby bovine kidney (MDBK) cell lineage.

*A. laidlawii* MDBK/IPV DNA was extracted from preparations of MDBK cells as follows: the supernatant of MDBK monolayers was clarified by low-speed centrifugation, filtered (0.45-µm pore size), and ultracentrifuged (6). DNA was extracted according to a phenol-chloroform protocol. Whole-genome paired-end sequencing was performed in the Illumina MiSeq platform (Illumina), with the 500-cycle kit (version 2). DNA libraries were prepared with a Nextera kit (Illumina). The original reads were imported into the Geneious software (version 8.1) and trimmed. The assembly of the *A. laidlawii* genome was accomplished by read mapping to the reference *A. laidlawii* genome (*A. laidlawii* PG-8A, GenBank accession no. CP000896) with the Geneious software and *de novo* assembly with the SPAdes assembler 3.6.0 (7). This yielded 12-fold average read depth. Gene prediction and annotation were performed automatically with the NCBI annotation tool (8). Statistics were generated by QUAST (9).

The genome of *A. laidlawii* MDBK/IPV consists of 29 contigs (largest contig, 212,101 bp; *N*_50_, 86,549 bp), with a total size of 1,307,942 bp. The G+C content of *A. laidlawii* MDBK/IPV is 31.97%. The *A. laidlawii* MDBK/IPV genome contains 1,250 predicted genes, two rRNA gene operons (5S-16S-23S), 33 tRNA genes, three noncoding RNAs (ncRNAs), and 25 pseudogenes. Among 1,183 potential protein-coding genes, 56% encode proteins with assigned functional roles. A high genetic repertoire was observed in *A. laidlawii* MDBK/IPV, which is described as necessary for adaptation to changing environmental conditions (5).

A comparative analysis between *A. laidlawii* MDBK/IPV and *A. laidlawii* PG-8A genomes shows the absence of approximately 200 kb in the MDBK/IPV genome. Interestingly, this PG-8A region is composed mainly of insertion elements (IS) and phage sequences. This genomic difference can be related to genome condensation or duplication of genetic material and integration events, which are commonly described in acholeplasmas (5).

The genome sequence of *A. laidlawii* MDBK/IPV will enable further investigations to better understand the molecular biology process of this bacterium that is important in culture cell contamination and in insights of minimal cellular life functions.

### Accession number(s).

The draft genome of *A. laidlawii* strain MDBK/IPV has been deposited at DDBJ/ENA/GenBank under the accession no. MIPS00000000. The version described in this paper is version MIPS01000000.
